# Supplementary material for: Differential Transcriptomic Profiles Following Stimulation with Lipopolysaccharide in Intestinal Organoids from Dogs with Inflammatory Bowel Disease and Intestinal Mast Cell Tumor
Source: Cancers (Basel). 2022 Jul 20;14(14):3525. doi: 10.3390/cancers14143525 (PMC9322748; doi:10.3390/cancers14143525)
Supplement: Supplementary file 1 [file cancers-14-03525-s001.zip › 5. Supplemental figure Legends.pdf]

Supplemental Figure Legends

Figure S1. Heatmaps (a) Heatmap of the differentially expressed genes in IBD enteroids; (b) Heatmap of the differentially expressed genes in IBD colonoids; (c) Heatmap of the differentially expressed genes in tumor enteroids.

Supplemental Table S1. Microarray analysis results. (a) LPS treated tumor enteroids versus Control tumor enteroids; (b) LPS treated IBD enteroids versus Control IBD enteroids; (c) LPS treated IBD colonoids versus Control IBD colonoids; (d) Control IBD colonoids versus Control IBD enteroids; (e) Control IBD enteroids versus Control tumor enteroids; (f) LPS treated IBD colonoids versus LPS treated IBD enteroids; (g) LPS treated IBD enteroids versus LPS treated tumor enteroids. The log-ratio M values represent log(R/G) (log fold change).
