# Supplementary figures and images for: Differential Transcriptomic Profiles Following Stimulation with Lipopolysaccharide in Intestinal Organoids from Dogs with Inflammatory Bowel Disease and Intestinal Mast Cell Tumor
Source: Cancers (Basel). 2022 Jul 20;14(14):3525. doi: 10.3390/cancers14143525 (PMC9322748; doi:10.3390/cancers14143525)

Color Key  
and Histogram

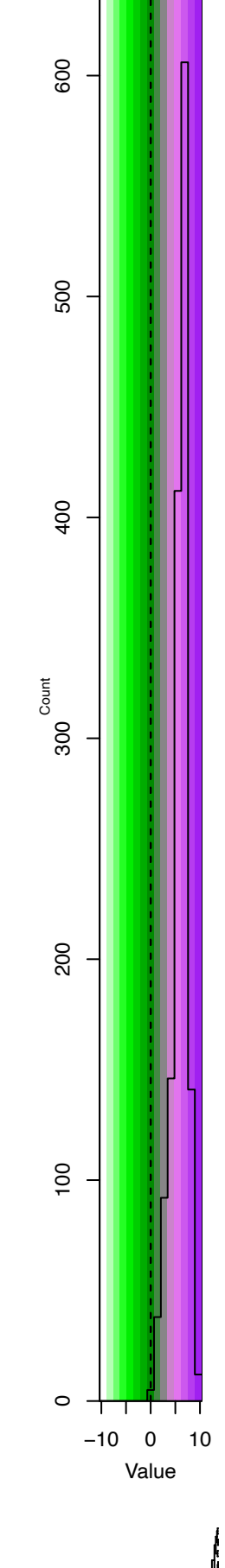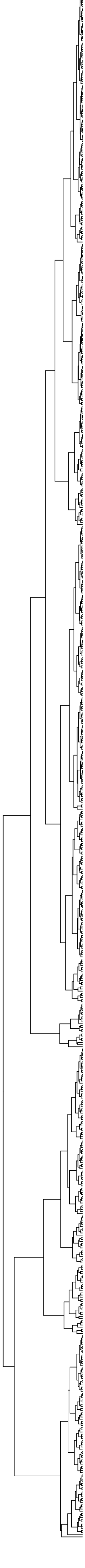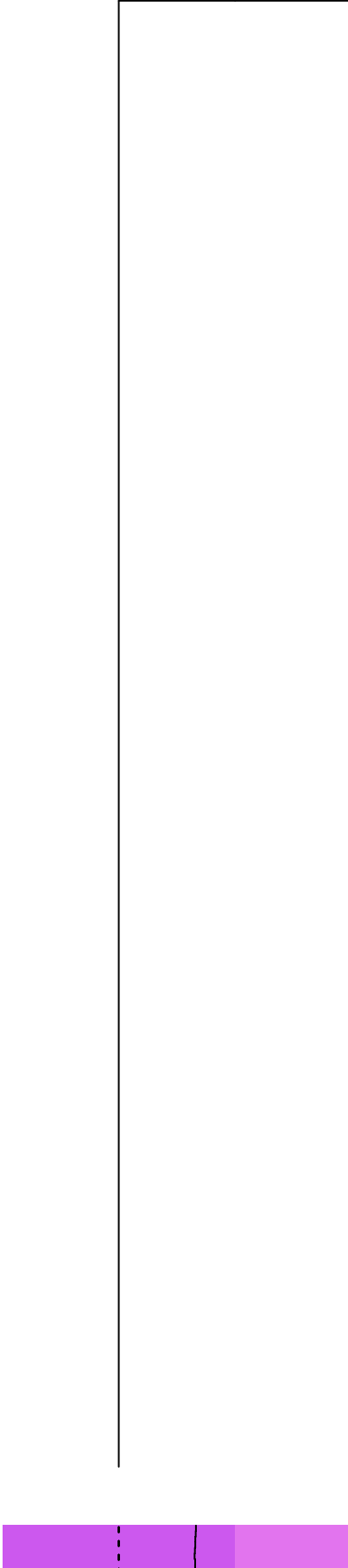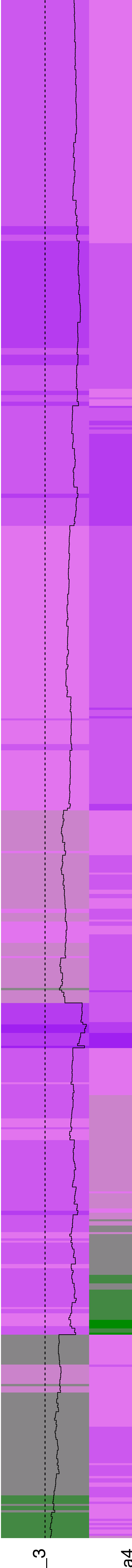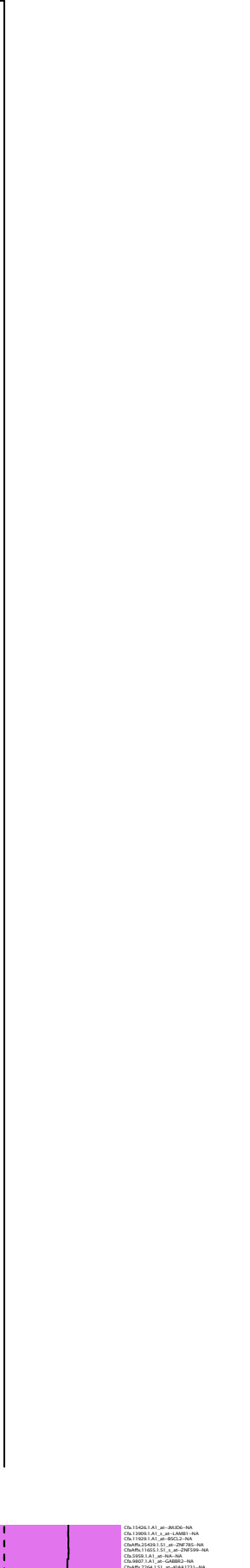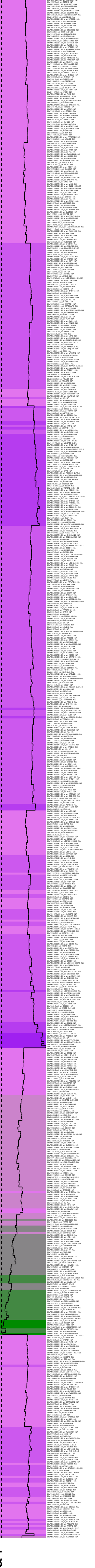

Supplement: Supplementary file 1 [file cancers-14-03525-s001.zip › 1. Supplemental Figure S1a-updated.pdf]

Color Key  
and Histogram

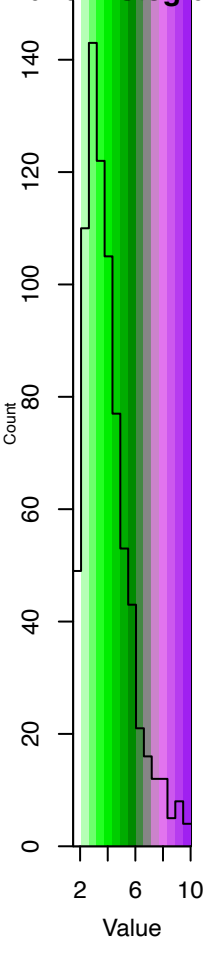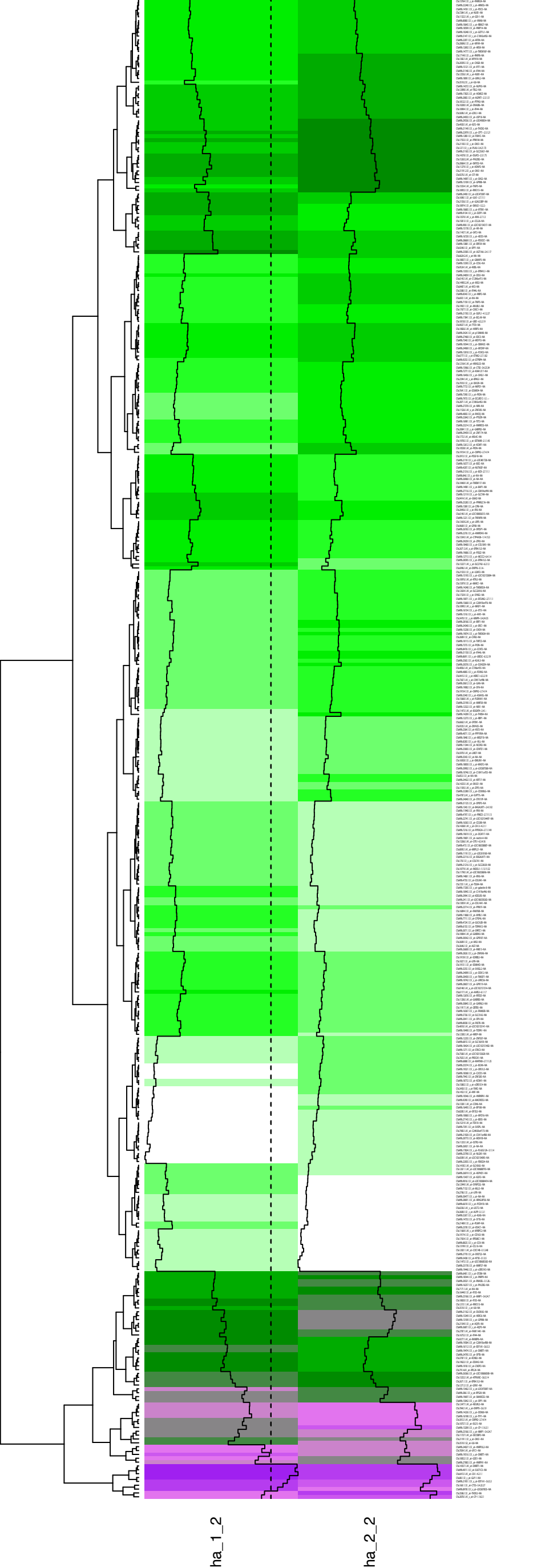

Supplement: Supplementary file 1 [file cancers-14-03525-s001.zip › 3. Supplemental Figure S1c-updated.pdf]
