# Supplementary material for: Differential Transcriptomic Profiles Following Stimulation with Lipopolysaccharide in Intestinal Organoids from Dogs with Inflammatory Bowel Disease and Intestinal Mast Cell Tumor
Source: Cancers (Basel). 2022 Jul 20;14(14):3525. doi: 10.3390/cancers14143525 (PMC9322748; doi:10.3390/cancers14143525)
Supplement: Supplementary file 1 [file 2. Supplemental Figure S1b-updated.pdf › 2. Supplemental Figure S1b-updated.pdf]

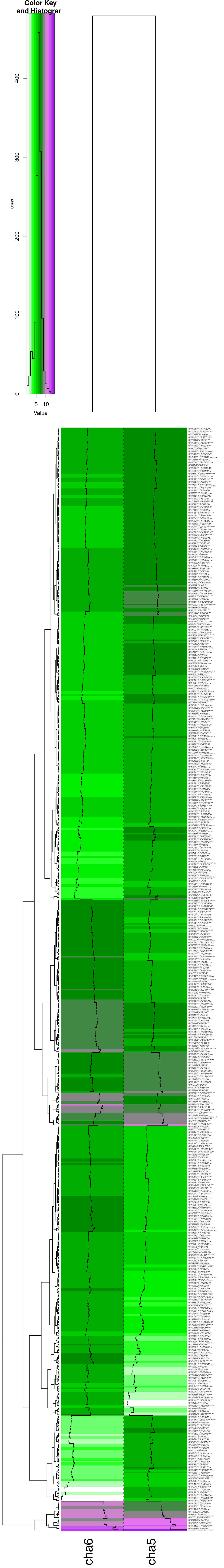
Color Key
and Histogram
Count
Value
400
300
200
100
0
5
10
cha6
cha5
